# A numerical framework for preprocedural prosthetic valve positioning and hemodynamic evaluation

**DOI:** 10.1007/s10237-025-02025-7

**Published:** 2025-12-12

**Authors:** Jonas Lantz, Jeremy D. Collins, Shuai Leng, Cynthia H. McCollough, Anders Persson, Tino Ebbers

**Affiliations:** 1 Department of Health, Medicine and Caring Sciences, Linköping University, Linköping, Sweden; 2 Center for Image Science and Visualization, Linköping University, Linköping, Sweden; 3 Department of Radiology, Mayo Clinic, Rochester, MN USA; 4 Department of Clinical Science, Intervention and Technology, Karolinska Institute, Stockholm, Sweden

**Keywords:** Aortic valve replacement, Computational fluid dynamics, Hemodynamics, Valve implantation planning, Cardiac CT, Medical image-based modeling

## Abstract

Aortic valve replacement is a cornerstone treatment for severe aortic valve diseases, including stenosis and regurgitation. Suboptimal valve seating can elevate the transvalvular pressure gradient, while valve orientation and size may produce flow jets that impinge on the ascending aorta, potentially weakening the vessel wall. Such hemodynamic complications can compromise valve performance and patient outcomes. This study presents a computational fluid dynamics framework, derived from medical CT images, for preprocedural hemodynamic assessment of aortic valve replacement. The framework minimizes user input and delivers rapid results, enabling efficient evaluation of valve types, orientations, and their hemodynamic impact. The results demonstrate that non-optimal implantation angles substantially increase pressure drop across the valve, thereby imposing higher workload on the heart. This automated and efficient simulation framework demonstrates strong potential for clinical application, supporting precise planning and execution of valve implantation procedures to improve patient care.

## Introduction

Aortic valve replacement has become a cornerstone in the treatment of severe aortic valve diseases, including aortic stenosis and aortic regurgitation. Over the past decades, the use of transcatheter aortic valve replacement (TAVR) has significantly increased, as it is a significantly less invasive alternative to traditional surgical aortic valve replacement (SAVR) (Nishimura et al. 2017; Mentias et al. 2021). Despite the success of TAVR, bioprosthetic valves have a finite lifespan and can fail over time due to degeneration, calcification, or structural damage (Dvir et al. 2014; Salaun et al. 2018). Depending on the type, size, and orientation of the implanted valve, increased valvular pressure gradients can occur (Midha et al. 2016), and the resulting high-velocity jet through the valve may impinge on the ascending aorta, a process that has been linked to progressive weakening of the aortic wall (Torii et al. 2013). These hemodynamic complications can adversely affect both valve performance and patient outcomes (Dvir et al. 2015).

Preoperative planning for TAVR procedures typically involves advanced medical imaging techniques, such as computed tomography (CT) or 3D echocardiography, to assess anatomical suitability and minimize procedural risks (Blanke et al. 2019). However, even with high-quality imaging, the optimal position and hemodynamic performance of the valve is difficult to predict prior to surgery. Computational fluid dynamics (CFD) based on medical imaging provides a solution by simulating blood flow through the heart and valves, thereby offering a detailed patient-specific estimation of hemodynamic conditions and performance of the implanted valve (Mittal et al. 2016; Lantz et al. 2021). We have previously demonstrated that intracardiac flow field patterns computed from CT show strong qualitative and quantitative agreement to the current reference standard, 4D Flow MRI (Lantz et al. 2018, 2021), confirming the robustness of the simulation approach. While CFD has been used to evaluate different valve positions and their effect on flow dynamics, shear stress, and regions at risk for thrombosis (Seo et al. 2020; Lantz et al. 2021; Lee et al. 2021; Asadi et al. 2022; Barrett et al. 2023; Brown et al. 2023; Crugnola et al. 2025), current patient-specific cardiac CFD models face several challenges. These include significant manual preprocessing and model creation and normally suffer from long compute times even on high-performance computer clusters.

A major challenge in traditional cardiac CFD methods is the reliance on structure-conforming computational grids to capture the complex structure and motion of the heart. The dynamic contraction and relaxation of the myocardium can cause folding or inversion of the computational grid, which ultimately makes the computation unable to continue. These challenges have been addressed by extensive re-gridding (Lantz et al. 2018, 2021; Bäck et al. 2024) during the cardiac motion, which increased the computational costs, or by significantly smoothing cardiac structures (Vedula et al. 2014; Chen et al. 2016; Gao et al. 2017; Asadi et al. 2022; Bennati et al. 2025), which may compromise model fidelity. Consequently, both model complexity and long computation times have hindered the translation of fluid flow simulations into clinical use.

An approach that overcomes most of the manual preprocessing steps of conventional CFD models is the immersed boundary (IB) method (Peskin 2002). This approach replaces the structure-conforming grid with a fixed Cartesian grid, incorporating the presence and motion of the structure via an additional force term in the governing equations. As a result, it eliminates the need for re-gridding during the complex cardiac contraction and relaxation, significantly streamlining the simulation process. Furthermore, the IB method facilitates easy integration of valve leaflet dynamics into a CFD model, allowing for a more comprehensive representation of the hemodynamic conditions. The IB method has previously been applied in cardiac studies to calculate both ventricular and valvular flow (Mittal et al. 2016; Vedula et al. 2016; Gao et al. 2017; Seo et al. 2020; Brown et al. 2023); however, prior implementations generally lacked the automation and streamlining required for efficient execution with minimal manual input. Enhancing the automation of these processes, while simultaneously making modeling choices that reduce computational demand further, could significantly improve the clinical applicability of CFD in cardiovascular care.

This study introduces an imaging-based IB CFD framework for preprocedural hemodynamic analysis of aortic valve replacement, with emphasis on evaluating alternative implantation orientations. The framework incorporates patient-specific cardiac structure and motion from time-resolved CT imaging, minimizes user involvement, and enables rapid turnaround times, thereby supporting systematic assessment of valve orientation and its hemodynamic impact.

## Methods

The computational framework was developed to evaluate hemodynamics across different valve types and dimensions while minimizing manual input and reducing computational demands. Patient-specific medical imaging of an existing aortic valve replacement was used to establish a baseline scenario, from which three hypothetical valve orientations were derived. Blood flow simulations were based on imaging data, and the required preprocessing steps are outlined below.

### CT acquisition and image processing

#### Patient characteristics and image acquisition

Institutional ethics board approval was obtained for image data to be retrospectively collected with waiver of informed consent from patients undergoing a clinically indicated coronary CT angiogram. Data were retrieved from a single patient (male, 84 years old) scanned on a dual-source photon-counting-detector (PCD) CT scanner (NAEOTOM Alpha, Siemens Healthineers). Retrospective ECG-gated scanning was performed without dose modulation, with the following acquisition parameters: tube voltage 120 kV, effective mAs 42, gantry rotation time 0.25 s, and 120 × 0.2 mm collimation.

Pitch was adapted to the patient’s heart rate. Images were reconstructed at every 5% between two R–R intervals, resulting in images from 0 to 95% of the cardiac cycle (i.e., 20 phases). The reconstructed slice thickness was 0.60 mm with a 0.30 mm increment, with an in-plane voxel size of 0.39 × 0.39 mm^2^. A Bv48 reconstruction kernel, 200 mm field-of-view, and 512 matrix size was used.

#### Image segmentation

**Fig. 1:**
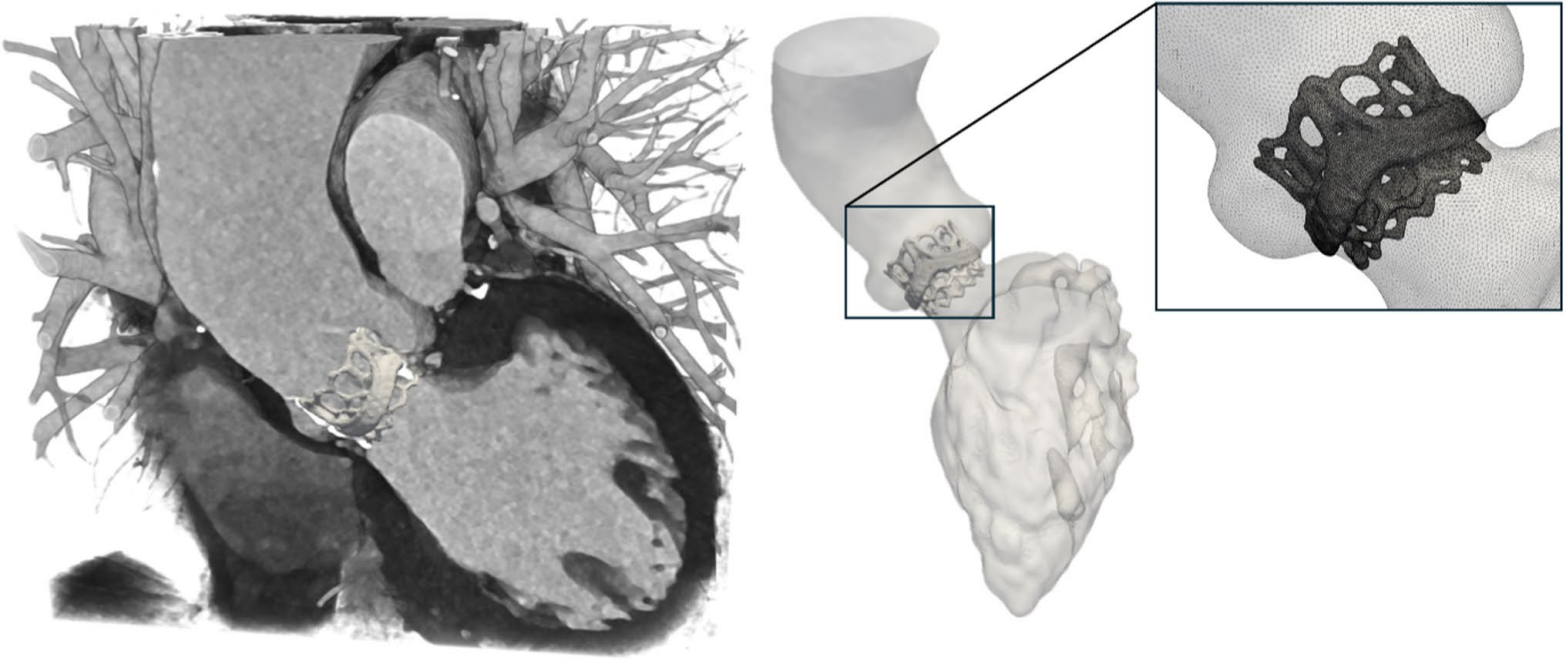
Left: volume rendering of the left heart from cardiac CT images together with the heart valve prosthesis. Right: geometrical representation of the left ventricle including papillary muscles and large-scale trabeculation of the ascending aorta and aortic heart valve. The inline figure illustrates the spatial resolution of the triangle mesh of the heart valve housing used in the simulations

The left ventricle (LV) and aorta were automatically segmented at end-diastole using an in-house AI software, with the cardiac geometry saved in STL-format for subsequent processing. For the LV, we retained the papillary muscles and large-scale trabeculation at the endothelial surface to preserve anatomical detail. The geometry was then imported into Gmsh v4.13 (Geuzaine and Remacle 2009) for surface regularization and remeshing into quadratic TRI6 elements with a target size of 0.5 mm. Similarly, the heart valve housing and leaflets were represented by 0.25 mm TRI6 elements to maintain as much of the geometrical detail as possible. A schematic view of the cardiac structures and valve mesh sizing is provided in Fig. 1

#### Cardiac motion

The motion of the segmented LV and aorta throughout the cardiac cycle was automatically extracted from the time-resolved CT data using a deformable image registration approach (Gupta et al. 2018). Specifically, a multi-resolution B-spline registration algorithm was applied between consecutive cardiac phases (20 phases over the R–R interval). The algorithm optimizes a similarity metric based on voxel intensities while imposing smoothness constraints on the deformation field, ensuring physiologically plausible displacements. The result is a dense displacement field defined for every voxel, from which the motion of all surface mesh points of the LV and aorta was interpolated. In this way, the full time-resolved wall motion was obtained for every point on the cardiac surface, without manual intervention. These wall motion fields were then directly used as prescribed input in the flow simulation framework. To obtain a smooth traverse in time from the 20 cardiac CT phases, the displacement field **D** of each point was interpolated in time using a piecewise cubic Hermite interpolating polynomial, as described in (Lantz et al. 2016). The accuracy of the extracted motion fields was evaluated by comparing deformed model surfaces against the original CT segmentations at end-systole, the phase of greatest deformation. Visual overlays (Appendix Fig. 9) demonstrate good agreement, indicating that the extracted motion reliably captures the CT-derived anatomy.

For the prosthetic valve, housing motion could not be prescribed directly from imaging data, as the CT acquisition only included the already implanted baseline configuration. Instead, a *landing zone* was defined as the annular region of the LV outflow tract in contact with the prosthetic valve housing for each implantation scenario. The rigid-body motion of this landing zone, extracted from the registered cardiac surfaces, was used to calculate valve housing motion for each virtual implantation. This approach enabled consistent evaluation of alternative valve orientations and positions without requiring direct image-derived motion of the prosthetic frame. Further details are provided in Sect. 2.4.1.

### Flow modeling

Based on the extracted cardiac motion from CT imaging, the intracardiac flow field was simulated using an IB method approach with an Eulerian description for the fluid and a Lagrangian description for the moving cardiac structure. Here, a fixed Eulerian domain Ω, with physical coordinates ${\boldsymbol{x}}=\left({x}_{1},{x}_{2},{x}_{3}\right)$ and a solid structure with reference coordinates ${\boldsymbol{X}}=\left({X}_{1},{X}_{2},{X}_{3}\right)$ are defined, where $\chi \left({\boldsymbol{X}},t\right)$ denotes the physical position of the material point ${\boldsymbol{X}}$ at time t. The system of equations can be expressed as follows:

1 $$\uprho \left( \frac{\partial \mathbf{u}\left(\mathbf{x},\mathrm{t}\right)}{\partial \mathrm{t}}+\mathbf{u}\left(\mathbf{x},\mathrm{t}\right)\cdot \nabla \mathbf{u}\left(\mathbf{x},\mathrm{t}\right)\right)=-\nabla p\left(\mathbf{x},\mathrm{t}\right)+\upmu {\nabla }^{2}\mathbf{u}\left(\mathbf{x},\mathrm{t}\right)+\mathbf{f}\left(\mathbf{x},\mathrm{t}\right)$$

2 $$\nabla \cdot \mathbf{u}\left(\mathbf{x},\mathrm{t}\right)=0$$

3 $$\mathbf{f}\left(\mathbf{x},\mathrm{t}\right)={\int }_{{\Omega }_{0}^{\mathrm{S}}}\mathbf{F}\left(\mathbf{X},\mathrm{t}\right)\updelta \left(\mathbf{x}-\upchi \left(\mathbf{X},\mathrm{t}\right)\right)\mathrm{d}\mathbf{X}$$

4 $$\frac{\partial\upchi }{\partial \mathrm{t}}\left(\mathbf{X},\mathrm{t}\right)=\mathbf{U}\left(\mathbf{X},\mathrm{t}\right)={\int }_{\Omega }\mathbf{u}\left(\mathbf{x},\mathrm{t}\right)\updelta \left(\mathbf{x}-\upchi \left(\mathbf{X},\mathrm{t}\right)\right)\mathbf{d}\mathrm{x}=\mathbf{u}\left(\upchi \left(\mathbf{X},\mathrm{t}\right),\mathrm{t}\right)$$

Equations (1) and (2) are, respectively, the momentum equation for a fluid and the conservation of mass, where ${\boldsymbol{u}}\left({\boldsymbol{x}},t\right)$ and $p\left({\boldsymbol{x}},t\right)$ denote the Eulerian velocity and pressure fields, while ${\boldsymbol{f}}\left({\boldsymbol{x}},t\right)$ represents an additional Eulerian force density used for the IB formulation. Equation (3) relates the Eulerian force density to the Lagrangian structure force density **F**(**X**,t) through the use of a Dirac delta function. In Eq. (4), ${\boldsymbol{U}}\left({\boldsymbol{X}},t\right)$ represents the Lagrangian structure velocity, enforcing the no-slip and no-penetration conditions on the fluid–structure interface.

To complete the system of equations, the Lagrangian structure force density is described using a penalty formulation, as:

5 $$\mathbf{F}\left(\mathbf{X},\mathrm{t}\right)=\upkappa \left(\left(\mathbf{X}-\upchi \left(\mathbf{X},\mathrm{t}\right)\right)+\mathbf{D}\left(\mathbf{X},\mathrm{t}\right)\right)-\upeta \left({\mathbf{U}}_{\mathrm{s}}\left(\mathbf{X},\mathrm{t}\right)-{\mathbf{U}}_{\mathrm{w}}\left(\mathbf{X},\mathrm{t}\right)\right)$$

Here, κ is a spring stiffness penalty parameter for the desired structure position, **D** the desired displacement of the cardiac structure position **X** at time t, and η is a damping term included for removing spurious numerical oscillations. **U**_**s**_ and **U**_**w**_ represent the computed wall velocity from image registration and the current wall velocity in the simulation, respectively. The desired displacement field **D** was extracted from the medical image data, as described in Sect. 2.2.1, while **U**_**s**_ was computed as the time rate-of-change of the structure position. The spring stiffness κ and damping factor η were set to 1e7 [Pa/m^2^] and 5e3 [Pa.s/m^2^], respectively. The spring stiffness and damping values were found to depend on several factors, including time step size and sizes of both the background grid and the cardiac structure (Lee and Griffith 2022). The numerical values of these parameters were obtained through a trial-and-error process, where optimal settings were identified when achieving the target wall displacement while avoiding numerical instabilities.

**Fig. 2:**
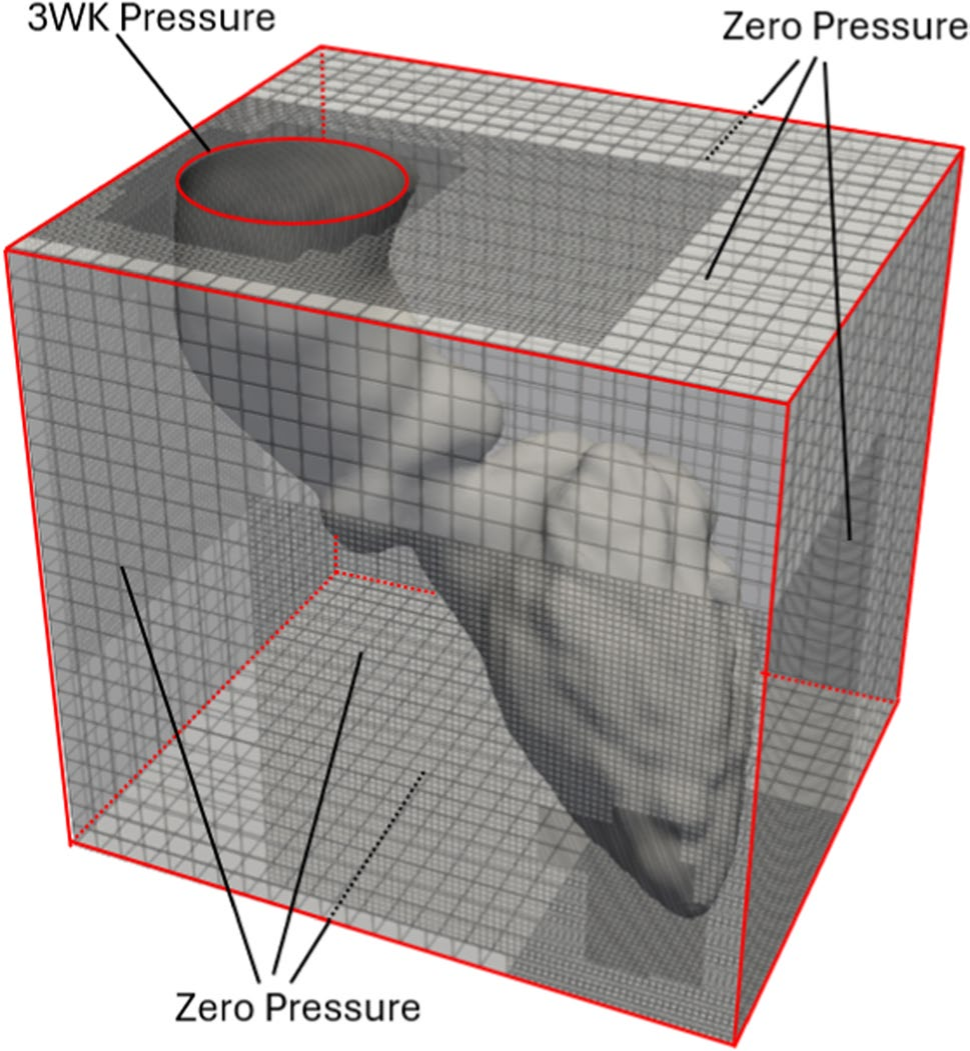
Eulerian (box) and Lagrangian (cardiac structure) domains. A three-element Windkessel (3WK) boundary condition was applied at the aortic outlet (red circle), while zero-pressure boundary conditions were applied at all other outer faces of the Eulerian grid. The figure also illustrates the three levels of adaptive mesh refinement, with the finest resolution located near the cardiac structures

The fluid properties were set to mimic blood, with a density of 1060 [kg/m^3^] and viscosity of 3.5e−3 [Pa.s]. Flow simulations were performed on a background grid using adaptive mesh refinement, with finer mesh cells placed within the heart to capture detailed hemodynamics, and coarser cells used outside the heart to conserve computational resources. Three levels of mesh refinement were implemented, each with a refinement ratio of 4, to adequately resolve the fluid flow dynamics, see Fig. 2. The finest level of mesh refinement encompassed the entire interior of the heart, with a background mesh size of 320 × 256 × 288 grid cells, yielding an effective fine scale isotropic resolution of 0.50 mm. This resolution is comparable, or even finer than, that employed in previous studies using the same IB method for cardiac applications (Griffith 2012; Kaiser et al. 2021, 2023). To ensure that results were not grid-dependent, a solution verification was performed on the Eulerian background grid spacing. Further details on this verification can be found in Appendix A.2. For the Lagrangian–Eulerian interaction described in Eq. (3), a three-point B-spline kernel was employed for the delta function. This choice was considered as the best all-purpose option of delta functions for both shear and pressure-dominant flows (Lee and Griffith 2022).

As the structure motion was prescribed from CT data and not part of a coupled fluid–structure simulation, the numerical stability of the simulation was less sensitive to the time step size. In this study, a fixed time step of 2.5e−5 [s] was employed, resulting in Courant–Friedrichs–Lewy (CFL) numbers generally in the range of 0.1–0.3, with no observed numerical instabilities. Zero-pressure boundary conditions were applied to all sides of the Eulerian grid, except at the aortic outlet, where a three-element Windkessel model was implemented, see Fig. 2. Since cardiac wall motion was prescribed, the aortic flow rate was known in advance, allowing the Windkessel parameters to be tuned to generate physiologically realistic pressure waveforms. The final parameters were R1 = 0.072 [mmHg·s/ml], R2 = 1.40 [mmHg·s/ml], and C = 0.88 [ml/mmHg]. Patient-specific blood pressure measurements were neither used nor required, as the simulation was driven entirely by prescribed motion and only relative pressures were needed. The outlet boundary was located distal to the valve jet, in a region where the main flow structures of interest had already developed, and the Windkessel model ensured physiological pressure waveforms while minimizing artificial reflections. As such, the use of this pressure-based boundary condition does not rely on a fully developed velocity profile at the outlet.

The fluid flow simulations were performed using the open-source software IBAMR (https://ibamr.github.io/) (Griffith et al. 2007; Griffith and Luo 2017). IBAMR uses SAMRAI (Hornung and Kohn 2002) for Cartesian grid discretization management, PETSc (Balay et al. 2019) for linear solver infrastructure, and libMesh (Kirk et al. 2006) for finite element representation.

### Prosthetic heart valve modeling

The patient studied here had a valve-in-valve (ViV) prosthesis consisting of a 23-mm Edwards SAPIEN transcatheter valve implanted within a 25-mm Edwards Magna surgical bioprosthetic valve. This ViV configuration was therefore used as the basis for all hemodynamic flow simulations. The framework itself is versatile and can accommodate other prosthesis types, sizes, or implantation strategies, including standalone TAVR and surgical bioprosthetic valves. In the current implementation, deployment mechanics of self- or balloon-expandable TAVR devices are not modeled; instead, the framework can incorporate the final deployed valve geometry obtained from post-procedural imaging or vendor specifications as input for hemodynamic analysis.

The valve motion within this framework was divided into two components: the rigid body movement (which encompasses both translational and rotational motion) driven by cardiac activity and the dynamics of the leaflets' opening and closing, which are influenced by the flow. The combination of these two components constitutes the valve's total motion, each of which is further described below.

#### Valve implantation and rigid body motion

**Fig. 3:**
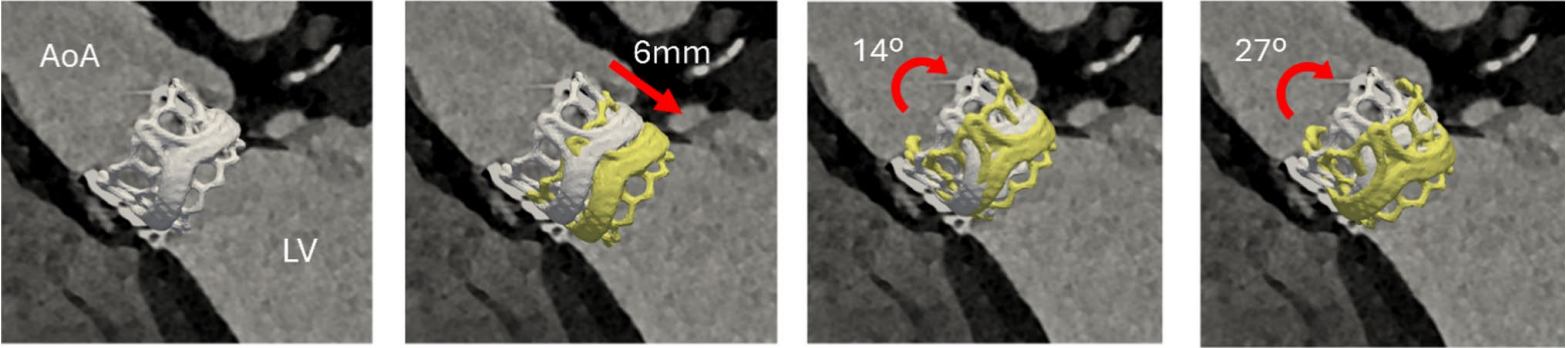
Virtual valve implantation. From left to right: baseline (in vivo), translated 6 mm ventricularly, rotated 14°, and rotated 27° around an axis lying in the aortic annular plane (orthogonal to the LVOT centerline). Baseline position shown in white; yellow surface indicates new valve position

In this framework, the heart valve housing was treated as a rigid structure that moved together with the surrounding cardiac tissue. A geometrical model of the heart valve was virtually implanted at four different locations: the baseline corresponding to the in vivo location, and additional positions where the valve was translated ventricularly 6 mm, and rotated 14° and 27° around an axis lying in the annular plane, orthogonal to the LVOT centerline, see Fig. 3. The selected implantation scenarios were designed to reflect clinically plausible deviations from the in vivo configuration. The 6-mm ventricular translation corresponds to variability in implant depth that can occur during positioning, while rotations of 14° and 27° represent increasingly misaligned orientations relative to the annular plane. The larger 27° case was included as a deliberately exaggerated scenario to test sensitivity of flow dynamics to severe malrotation. To ensure full apposition and avoid artificial paravalvular leakage, the annular surface was contracted by a small margin around the valve housing so that contact was always maintained in the simulations. The valve landing zone was defined as the annular region of the LV outflow tract in direct contact with the prosthetic housing, identified using a nearest-neighbor approach. The average motion of this region was determined from the image registration fields, and Horn’s quaternion-based algorithm (Horn 1987) was used to compute the optimal rigid-body transformation that maps the landing zone motion onto the prosthetic valve housing and its leaflets. In this way, the prosthetic valve was coupled to the prescribed motion of the cardiac structures while remaining undeformed during the simulation. Horn’s algorithm is a widely adopted method for calculating the optimal rigid transformation that aligns two sets of 3D points. In brief, the method computes the cross-covariance matrix of the two 3D point datasets and identifies the optimal quaternion rotation using singular value decomposition.

#### Valve leaflet dynamics

The dynamics of the valve leaflets during opening and closing were simulated using a reduced degree-of-freedom approach in which the transvalvular pressure gradient directly governed leaflet motion. This approach follows the general strategy of previously published reduced-order models (Domenichini and Pedrizzetti 2015; Bailoor et al. 2021; Zhu et al. 2022), but it is described in detail here. The leaflets were segmented from the CT imaging data in their closed position and were then manually deformed into the fully open position using MeshMixer (v3.5, Autodesk Inc.). By maintaining constant topology throughout the deformation process, a one-to-one point correspondence was established between all points on the leaflets in both the open and closed positions. This correspondence facilitated the precise prescription of the opening and closing states during the flow simulations.

**Fig. 4:**
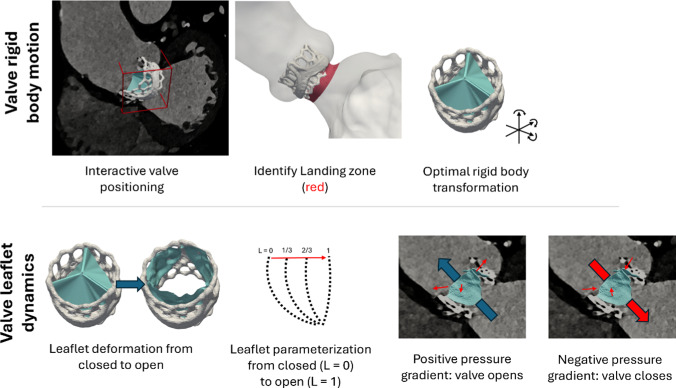
Upper panel: The prosthetic heart valve was virtually implanted using CT images for alignment. Then, the contact area between the cardiac structure and the valve—the landing zone—was extracted and used as input for an alignment algorithm to obtain the optimal rigid-body motion of the valve. Lower panel: The valve leaflets were segmented in the closed position and deformed into the open position. The leaflet position was then parameterized from closed to open, and in the flow simulation, their motion was determined by the valvular pressure gradient

During the flow simulation, the valve leaflet opening and closing dynamics were governed by the valvular pressure gradient. When the pressure gradient was positive in the general flow direction (from LV to aorta) and exceeded a threshold *P*_open_, the valve leaflets transitioned from the fully closed to the fully open position at a fixed rate *R*_leaf_ = 1/time_open_, where time_open_ is a predetermined duration for this transition. In this model, the closed position is represented as *L* = 0 and the fully open as *L* = 1. The valve position on the interval *L* = [0,1] was then computed at each time step in the flow simulation as *L* = *L* + *R*_leaf_*Δ*t* where Δ*t* is the simulation time step. Conversely, if the valvular pressure gradient fell below a threshold P_close_, indicating that the leaflet should close, the dynamics reverted to a closed state, and the position was updated as *L* = *L* − *R*_leaf_*Δ*t*, see Fig. 4.

In vivo, opening and closing of prosthetic valve leaflets generally occur within 25–75 ms (Luraghi et al. 2018; Lee et al. 2020; Seo et al. 2020; Bailoor et al. 2021; Govindarajan et al. 2022; Brown et al. 2023), with these durations varying based on factors such as material stiffness, degree of calcification, and valvular pressure gradient. In the absence of specific in vivo data, *R*_leaf_ was set to 20 [1/s], which corresponds to an opening and closing time of 50 ms. A sensitivity analysis of the opening and closing rate *R*_leaf_ was performed, see Appendix A.3. The pressure differences required to initiate opening or closing of the valve, *P*_open_ and *P*_close_, were both set to 1 [mmHg]. This small threshold was not intended to represent inertia or frictional effects, but rather to prevent instantaneous switching at zero gradient and to ensure numerical stability.

This approach employs a reduced-order model designed to minimize computational cost; it does not account for valve inertance (Casas et al. 2017) or leaflet fluttering (Bailoor et al. 2021). Nevertheless, reduced-order formulations of this type have been widely applied in CFD studies of valve hemodynamics, and in combination with sensitivity analysis provide a reliable and computationally efficient approximation of prosthetic valve leaflet motion.

### Evaluation

For qualitative evaluation, snapshots of the velocity magnitude were visualized in 3D and on cross-sectional planes of the ascending aorta. These visualizations provide an intuitive understanding of the flow field and help assess the impact of different heart valve orientations on the flow. The velocity magnitude reveals insights into the aortic flow jet, including its trajectory and potential impingement against the aortic wall, which may influence flow patterns and hemodynamic efficiency.

Quantitatively, the pressure drop across the valve is an important metric, as elevated values may induce myocardial remodeling and ultimately lead to heart failure. Clinically, this metric can be obtained through invasive catheter-based pressure measurements or estimated from velocity profiles using echocardiography. In this study, the time-varying pressure drop was directly obtained from the simulation. The pressure in the LV and ascending aorta were sampled and averaged from point clouds, each consisting of 20 points.

Viscous dissipation is a measure of fluid kinetic energy lost to heat due to high shear gradients, and when integrated over a volume, it represents the associated power loss *P*_loss_:

$${P}_{\mathrm{loss}}={\int }_{V}2\upmu {{\boldsymbol{S}}}_{ij}:{{\boldsymbol{S}}}_{ij }{\mathrm{dV}}$$

where µ is the fluid viscosity, and **S**_ij_ is the rate-of-deformation tensor, with the integration performed over both the LV and ascending aorta. An additional integration over time gives the associated energy loss *E*_loss_:

$${E}_{\mathrm{loss}}={\int }_{T}{P}_{\mathrm{loss}}{\mathrm{dt}}$$

Both metrics were evaluated to identify elevated levels, which may indicate a suboptimal valve orientation. All reported hemodynamic results were from the second cardiac cycle, after the first cycle was discarded as initialization to allow periodicity to be established.

### Automated processing framework

An automated framework was implemented as a modular backend, where each stage (image segmentation, image registration, geometry processing, surface meshing, fluid solver input generation, and fluid flow simulation) was implemented as a separate containerized module. A central Python-based controller managed the execution flow, read user-specified configuration files, and passed standardized data structures between modules.

Valve positioning was left under user control, enabling direct testing of clinically relevant placement scenarios. This was accommodated in two ways. In interactive mode, the framework paused after the segmentation module and waited for user-provided positioning, after which preprocessing resumed automatically. Alternatively, multiple candidate placements could be defined in the initial configuration file, in which case the framework generated solver input files for each scenario and submitted the corresponding jobs in parallel. This design enabled both single-case studies and systematic exploration of implantation strategies with minimal additional effort.

Once segmentation was available, the controller sequentially executed all modules to generate solver input files and organize outputs in a standardized directory structure, after which jobs were submitted to the HPC system. Intermediate results were stored in a hierarchical directory system with unique case identifiers, and metadata files linked outputs to the original CT dataset and chosen parameters. Configuration files defined all module parameters, and all runs were logged with execution time, parameter values, and software versions, ensuring reproducibility and comparability across scenarios.

## Results

### Processing and run times of simulation framework

All main preprocessing steps of the simulation framework were automated and executed without manual interaction. Using only clinically available CT data as input, segmentation, image registration and writing input data for the flow simulation took approximately 45 min on a standard workstation. The flow through the heart valve was a direct consequence of the ventricular contraction, which in turn, was prescribed from the CT imaging data. When preprocessing was finished, the framework was halted and allowed for interactive implantation and positioning of the prosthetic aortic valve in the segmented cardiac geometry. When satisfied with the orientation, the simulation framework resumed and first computed the rigid body motion of the implanted aortic valve from the landing zone motion, which took less than 1 min. Then, the fluid flow simulation was initialized. Compute time for the flow simulations on 32 CPU cores with 64 GB RAM took on the order of 8–10 h per cardiac cycle with the chosen background grid resolution and time step size. While it was possible to run the flow simulations on a powerful workstation, in practice the simulations were performed on a single compute node at the National Supercomputer Center (NSC) at Linköping University.

### Aortic flow field

**Fig. 5:**
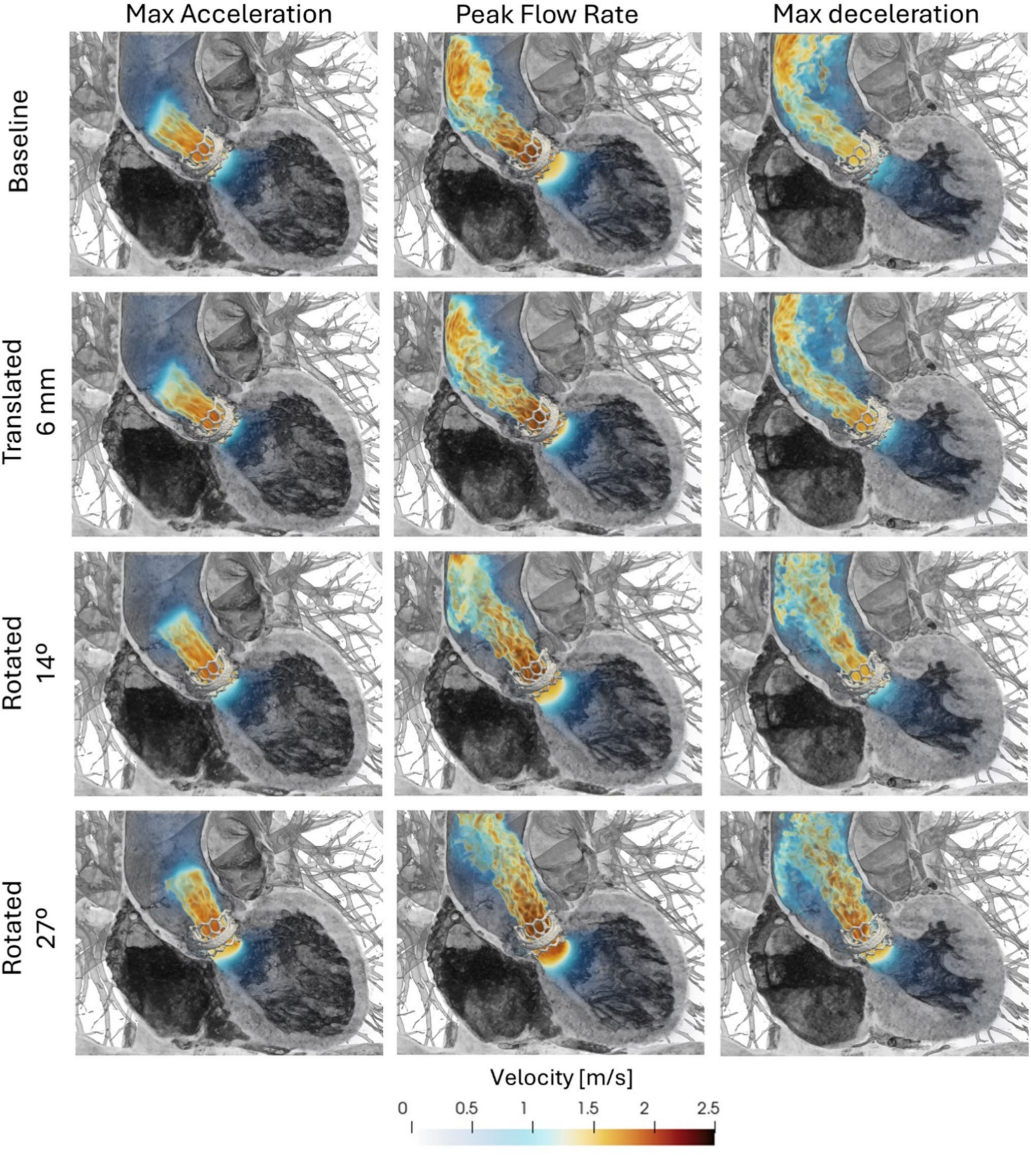
Volume rendering of velocity at maximum flow acceleration, peak flow rate, and maximum flow deceleration for the four different valve orientations. Regions of low velocity are not shown to improve visualization of major flow structures, and the valve leaflets have been omitted in the renderings for clarity

To qualitatively assess the impact of different valve orientations on the aortic flow field, volume renderings of the velocity magnitude through the LV and ascending aorta were visualized (Fig. 5). The flow field was visualized at three key phases: maximum acceleration, peak flow rate, and maximum deceleration.

In the baseline case, representing in vivo conditions, the narrowing of the valve created a flow jet with peak velocities of approximately 2.5 m/s that impinged on the outer curvature of the ascending aorta before being deflected toward the aortic arch. In the case where the valve is translated 6 mm ventricularly, a qualitatively similar flow jet structure was observed as in the baseline case. This is intuitively expected, as the valve’s translation does not alter the direction of the flow jet. For both the baseline case and translated cases, the flow near the inner curvature of the ascending aorta comprised a low-speed region.

At a 14° rotation, the aortic flow jet maintained similar peak flow velocities but no longer directly impacted the outer curvature of the ascending aorta. Instead, the flow jet was directed more centrally within the ascending aorta before it started to dissipate. In the case of a 27° rotation, the aortic flow jet instead approached impingement on the inner curvature of the ascending aorta, as the valve displaced the flow jet toward the left atrium.

**Fig. 6:**
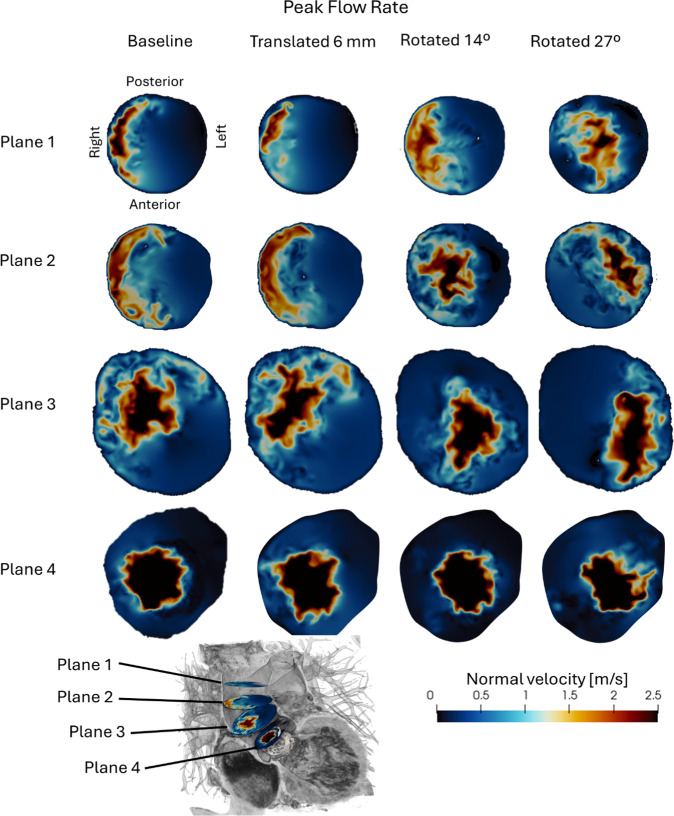
The velocity magnitude normal to planes in the ascending aorta for the four valve orientations at peak flow rate

To further assess the effects of valve implantation orientation on the aortic flow, the velocity magnitude normal to slice planes perpendicular to the ascending aorta was computed, see Fig. 6. These slice planes allowed for a detailed visualization of the flow structure at specific locations along the aorta, with the normal velocity component plotted to highlight the flow direction and magnitude.

In Fig. 6, slice planes covering the ascending aorta depict the normal velocity at peak flow rate. The upper left contour plot illustrates the anatomical orientation of these planes, with Plane 1 representing the most distal section in the ascending aorta. In the baseline case, the jet is clearly displaced toward the right side, which aligns with the observed impingement of the flow on the outer curvature. This displacement is indicative of an eccentric flow pattern, which may increase chronic hemodynamic loading on the outer aortic wall and has been associated with aortic dilatation. For the valve translated 6 mm toward the ventricle, a similar flow pattern to the baseline case is observed, with the jet still directed toward the outer curvature. The consistent flow behavior suggests that a mere translation in the flow direction does not substantially change the jet trajectory, as the general direction of the blood flow remains unaffected. In contrast, the 14° rotated valve orientation results in a more centralized flow profile, as evident in slice planes 2 and 3. The 27° rotated valve orientation exhibits a distinct flow pattern, with the jet directed toward the left-inner curvature of the ascending aorta.

### Pressure drop and viscous losses

**Fig. 7:**
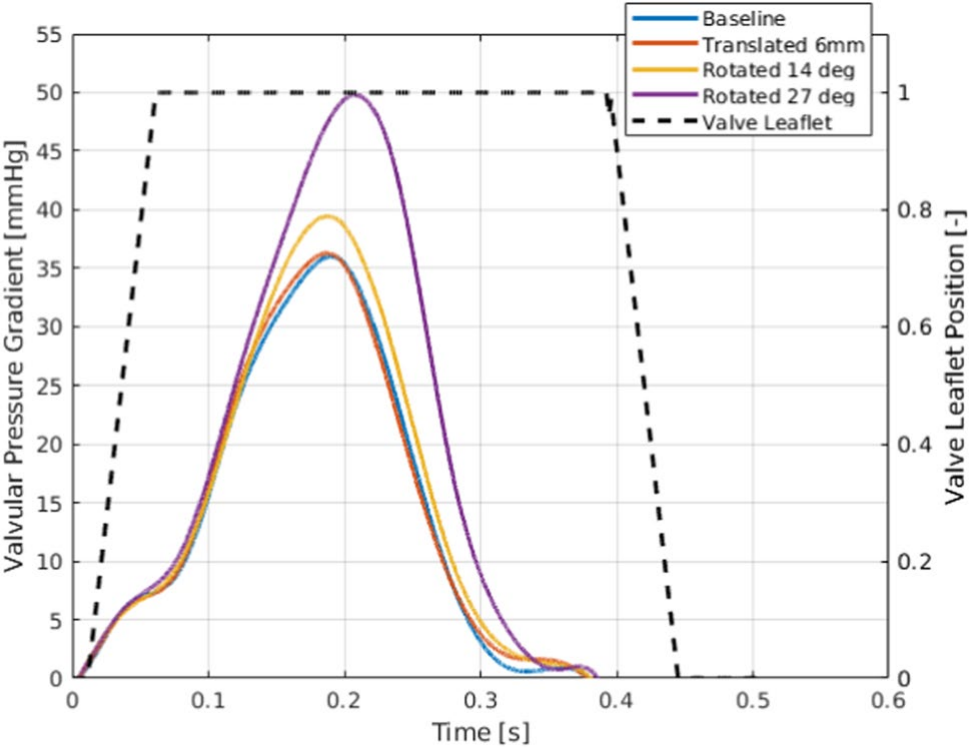
The valvular pressure drops for the four different valve orientations (left y-axis) and valve leaflet position (open/closed, right y-axis)

The influence of valve orientation on the pressure drop across the valve was evaluated, with the results plotted in Fig. 7.

For the first three configurations, the pressure drop remained relatively consistent, with an instantaneous peak value of approximately 35–37 mmHg. However, for the case with the valve rotated by 27°, a substantially higher pressure drop was observed, reaching a maximum of 50 mmHg. This increased pressure drop reflects a less optimal flow condition, potentially due to the altered trajectory of the aortic jet, as mentioned earlier.

The valve leaflet dynamics are also depicted in Fig. 7, where the position ranges from 0 (fully closed) to 1 (fully open). All four cases exhibited similar opening and closing behaviors, with the leaflets responding to the valvular pressure gradient in the same manner. The prescribed opening and closing duration of 50 ms was consistent across cases, aligning with physiological expectations for prosthetic valve dynamics. The leaflets initiated opening when the pressure gradient was positive and exceeded 1 mmHg, while closure commenced when the gradient was negative and below − 1 mmHg. For completeness, Appendix Fig. 13 shows static pressure fields relative to the outlet pressure. These plots demonstrate the higher LV pressures associated with increased transvalvular resistance, while also confirming that the jet cores correspond to low static pressure regions in the ascending aorta.

**Fig. 8:**
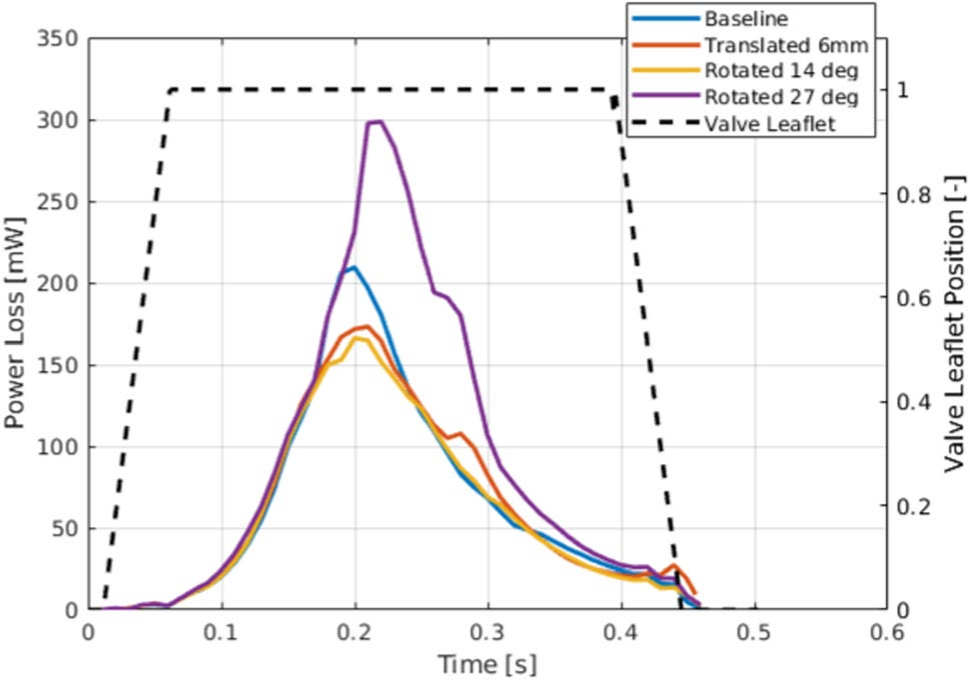
Time course of the power loss from viscous dissipation for the four different simulation cases. Dashed black line indicates valve leaflet position (0 = fully closed, 1 = fully open)

Additionally, Fig. 8 and Table 1 compare the power and energy loss associated with each valve orientation. For the first three cases, energy losses were similar, ranging from 27 to 29 mJ. This suggests that despite slight variations in flow patterns, the dissipation of energy due to viscous effects remained relatively stable. In contrast, the 27° rotated valve exhibited a markedly higher energy loss of 40 mJ, which can be attributed to the less favorable flow field observed in this configuration.

**Table 1:** The total energy loss for each evaluated implantation case

Valve case	Energy loss (mJ)
Baseline	29.1
Translated 6 mm	28.8
Rotated 14°	27.0
Rotated 27°	40.1

## Discussion

This study presents a numerical framework designed to compute the hemodynamic impact of different orientations of bioprosthetic aortic valve implants. We demonstrated a fully automated simulation pipeline with low computational demands, supporting future integration into clinical workflows for patient-specific planning. Our results indicate that suboptimal implantation angles can significantly increase the valvular pressure drop, thereby increasing the hemodynamic load on the heart.

### Numerical framework and possible translation into clinical practice

The simulation framework presented in this study demonstrates a high degree of automation during preprocessing, which is an essential feature for clinical applicability, where minimal manual intervention is preferred. The fully automated pipeline from CT data to simulation-ready models supports the potential for integration into patient-specific planning workflows. This capability could enable clinicians to evaluate various valve implantation strategies in advance, optimizing orientation and placement prior to the actual procedure.

#### Automation and modeling choices

To streamline the workflow and reduce user interaction, the modeling decisions aimed to automate the process while maintaining low computational costs. The most computationally intensive part remained the flow simulations, accounting for approximately 90% of the total runtime. The use of a prescribed motion approach, where cardiac motion is based on medical image data rather than coupled fluid–structure interaction (FSI) modeling, was a deliberate choice to reduce both simulation runtime and model complexity. Previous studies using coupled FSI models have reported runtimes extending up to several days or even a week on high-performance computing (HPC) systems for a single cardiac cycle (Gao et al. 2017; Kaiser et al. 2023), whereas our approach achieved flow results within half a day on a high-end workstation. Contrary to the previous studies, our numerical framework also included the contraction and relaxation of the ventricle, providing patient-specific flow rates through the aortic valve. While the presented approach significantly reduces computational costs and enables faster turnaround times, it does not capture all fluid–structure interaction (FSI) effects present in more computationally expensive models.

The decision to use an image-based model for ventricular motion offered several advantages. With this approach, the aortic flow rate is known prior to performing the flow computation, enabling efficient tuning of pressure boundary conditions. Moreover, using clinical image data, we already have the ground truth patient-specific cardiac contraction patterns, and we can impose it directly, which increases the fidelity of the model. Since the motion is extracted from time-resolved imaging, the heart rate is inherently embedded in the data through the temporal resolution and frame rate of the acquisition. As such, the simulated hemodynamics naturally reflect the patient’s heart rate at the time of imaging. While this method does not treat heart rate as an independently tunable parameter, alternative datasets with different heart rates could be used to explore rate-dependent phenomena. In addition, time-scaling of the motion profile, allowing differential adjustment of the systolic and diastolic phases, could approximate the hemodynamic effects of varying heart rates. This could be, particularly, valuable in assessing device performance across arrhythmic conditions or during pharmacological interventions affecting heart rate. In contrast to image-based models where cardiac motion is prescribed, electrophysiological models for muscle contraction require additional assumptions and are often unable to capture specific anatomical details like papillary muscle motion or wall thickening, which can influence intraventricular flow dynamics (Lantz et al. 2018).

While fluid–structure interaction (FSI) models of native and prosthetic valves are gaining more and more attention due to their ability to couple leaflet motion to the hemodynamics, stress evaluation, and even leaflet fluttering (Gao et al. 2017; Lee et al. 2021; Brown et al. 2023; Fumagalli et al. 2023; Kaiser et al. 2023), they also require detailed information on material models and fiber directions, which can be highly patient-specific. In this study, we chose a reduced-order approach for leaflet motion, similar to (Bailoor et al. 2021) to capture the large-scale motion of the leaflets dynamics and their impact on hemodynamics. An additional feature of our approach is that the rigid-body motion of the valve could be accounted for, as the ventricular and aortic motion was imposed into the simulation.

#### Implications for clinical implementation

The simulation framework demonstrated a high degree of automation in the preprocessing steps, which is crucial for clinical applications where manual intervention needs to be minimized. This rapid turnaround from imaging to simulation setup allows for potential future applications in patient-specific planning and clinical decision-making. The ability to interactively adjust valve positioning within the framework provides flexibility and can accommodate various clinical scenarios, such as simulating multiple valve orientations before an actual procedure to determine the optimal implantation strategy. While the framework focuses on different orientations in valve replacement, numerical modeling is also expected to play an important role in valve repair (Wong et al. 2023).

A clinically important future direction is to use patient-specific anatomy and evaluate pre- and post-implant hemodynamics. Modeling a malfunctioning baseline valve, e.g., a case with leaflet degeneration, could offer a more clinically relevant reference. Although prosthetic leaflet motion is difficult to visualize in CT due to limitations in temporal resolution, reduced leaflet mobility or increased stiffness could be incorporated parametrically in the model to simulate valve dysfunction. Additionally, surgical suturing can alter the annular geometry, which may affect both valve seating and flow. Future efforts may include modeling such annular distortion or incorporating biomechanical deformation to better capture post-surgical anatomy.

For transcatheter aortic valve replacement (TAVR), additional considerations arise since the deployed geometry depends strongly on patient-specific anatomy and the mechanics of self- or balloon-expansion. The present framework does not simulate deployment mechanics, but it can incorporate the final valve geometry once known, either from post-procedural imaging or from vendor specifications. This enables evaluation of hemodynamics under realistic boundary conditions even without explicit expansion modeling. In the future, coupling the framework to finite-element models of stent deployment or anatomy-driven expansion predictions could allow fully predictive TAVR simulations, bridging the gap between deployment mechanics and post-implant flow analysis.

Alternative approaches can prescribe inflow conditions from Doppler ultrasound or 4D flow MRI, and these modalities may be appropriate when available, particularly for simplified LVOT–aorta models. However, ultrasound is generally limited to velocity estimates along a single axis, and MRI may not always be feasible in patients with prosthetic valves. In this study, the availability of 4D CT enabled us to prescribe patient-specific wall motion of both the LV and aorta, thereby capturing intraventricular flow patterns and LV—aortic coupling beyond what can be achieved from inlet velocity profiles alone.

Looking ahead, integrating this modeling framework with intraoperative guidance tools, such as augmented reality (AR) overlays, could help translate pre-surgical planning into precise implantation. While applying such guidance during open surgery remains challenging, it is more feasible for catheter-based procedures like TAVR, where real-time AR could potentially assist in replicating the preoperatively simulated optimal configuration. It should be emphasized that the present study is not intended as a direct surgical guideline, but rather as a methodological framework for systematically exploring implantation scenarios in silico*.*

### Hemodynamic evaluation of valve orientation

Implanting a prosthetic heart valve in the exact desired orientation and location is challenging, and an in vitro study has previously shown that optimal positioning may be different between different valve types (Midha et al. 2016). In this study, we evaluated different valve configurations, including a baseline in vivo position, a 6 mm translation along the flow direction, and two rotations (14° and 27°) in the atrioventricular plane. Both qualitative and quantitative analyses were used to assess how these variations influence aortic flow and pressure dynamics.

The tested configurations reflect common variations observed in clinical practice. First, valves are often sewn off-center due to anatomical constraints, particularly in patients with large annuli or extensive calcification. Second, tilting of the valve, whether intentional or not, can alter the trajectory of the ejected flow. Third, implanting a smaller valve than the native annulus is common, especially during re-interventions, as downsizing is technically easier than upsizing. All these scenarios can affect transvalvular pressure gradients and downstream flow patterns in clinically significant ways.

Our simulations indicate that these geometric deviations can increase pressure gradients and redirect jet trajectories, leading to altered wall impingement patterns. These findings reinforce the importance of individualized preoperative planning to evaluate the patient-specific hemodynamic effects of valve orientation.

#### Qualitative analysis: flow patterns

Flow patterns varied distinctly across configurations. In both the baseline and translated cases, the flow jet impinged on the outer curvature of the ascending aorta and deflected toward the arch, suggesting that translation alone had limited influence on flow direction. In contrast, the 14° rotated valve yielded a more centralized jet within the lumen, allowing the flow to dissipate energy more uniformly before contacting the aortic wall. The 27° rotation, however, redirected the jet toward the inner curvature of the ascending aorta, and this change in jet trajectory could lead to concentrated high-velocity regions and increased stresses, which are associated with adverse remodeling of the aortic wall and increased risk of pathological changes such as aortic dilation.

#### Quantitative analysis: pressure drops and losses

Quantitatively, the pressure drops across the valve were similar for the baseline, translated, and 14° rotated cases, ranging between 35 and 37 mmHg. However, the 27° rotation resulted in a significantly higher pressure drop of 50 mmHg. This increase indicates that an unfavorable valve orientation can substantially elevate the pressure gradient across the valve, which may contribute to higher myocardial workload and potential heart failure. The results emphasize the importance of aligning the valve with the main flow direction to minimize pressure gradients.

The analysis of viscous power and energy losses further supported these findings. While the baseline case showed slightly higher viscous losses than the translated configuration, the 27° rotated valve exhibited the highest energy loss (40 mJ compared to 27–29 mJ in the other configurations). This suggests that the 27° rotation induced flow patterns with greater shear gradients, resulting in more kinetic energy being converted to heat and, consequently, higher hemodynamic inefficiency.

#### Clinical implications of valve orientation

The findings underscore the sensitivity of valve orientation to hemodynamic performance. While minor variations in valve positioning did not significantly affect pressure drop or leaflet dynamics, certain configurations—especially the 27° rotation—had a marked impact on flow efficiency. The 27° rotation may have introduced localized regions of high velocity and shear stress, contributing to increased mechanical stress on the aortic wall and higher workload for the heart. Centralized flow patterns, as seen in the 14° rotated case, are typically associated with improved hemodynamic efficiency. This configuration may help distribute stress more evenly across the vessel wall, potentially mitigating the risk of adverse remodeling and other pathological outcomes.

The results suggest that while small changes in valve orientation may not drastically alter leaflet dynamics or pressure gradients, optimizing valve alignment is crucial for minimizing energy losses and improving overall flow patterns. Careful consideration of valve implantation angles during surgery could enhance long-term outcomes for patients undergoing aortic valve replacement by reducing hemodynamic inefficiency and the associated risk of elevated pressure drops and stress on the aortic wall.

### Limitations

The use of a prescribed motion model instead of a fully coupled fluid–structure interaction (FSI) approach offers advantages such as ease of setup, reduced dependency on modeling assumptions, and shorter computation times. However, this choice comes with limitations, primarily because the model does not account for the dynamic changes in myocardial function or remodeling due to increased workload. Since changes in hemodynamics do not influence the ventricular volume using this approach, modeling the Frank–Starling mechanism is not possible and both short- or long-term ventricular remodeling cannot be directly inferred from CT data alone. Although relying on clinical imaging data for patient-specific motion improves the accuracy of the simulation, the inability to model active muscle contraction and its effect on hemodynamics remains a limitation.

The IB method allows for flow simulations with significant structural deformation without the need for remeshing, making it a good choice for cardiac applications. However, due to the nature of the current IB method and implementation, it does not currently allow for accurate computation of shear stresses at surfaces, such as the heart valve or aorta. However, the advantages of not needing to spend significant time on remeshing due to the complex motion of the contracting and relaxing LV outweigh this limitation.

The quantitative evaluation was limited to pressure drop and energy loss, which are widely used in the prosthetic valve literature. Pressure drop is directly measurable in the clinic, whereas energy loss is a derived metric that complements pressure drop by integrating flow information to assess overall hemodynamic efficiency. We acknowledge that additional descriptors, such as wall shear stress distributions or vortex quantification, may provide further insights into flow-induced remodeling and device performance. These advanced metrics were not included in the present work but represent an important direction for future studies.

Additionally, the simulation assumes no mitral regurgitation or ventricular septal defect, both of which could alter intraventricular flow patterns and should be considered in future work aimed at broader patient populations. Furthermore, the impact of valve deployment on cardiac structure and motion was not simulated in this study. Deployment-related changes could influence the structural dynamics of the heart and affect both valve function and orientation. Given that the study focused on hemodynamics, these aspects were beyond the scope of the current work but represent areas for future research. Finally, the present study was not validated against patient-specific Doppler echocardiography or catheter-based pressure data, which will be an important step in future work to confirm clinical applicability.

## Conclusion

By streamlining the computational process and incorporating advanced imaging data, our framework bridges the gap between research and clinical application. Integrating clinical imaging data into the numerical framework enables visualization of patient-specific flow patterns and optimization of valve placement. The ability to interactively assess hemodynamic outcomes from a model with a high degree of automation and relatively low computational demands offers a powerful means to improve patient outcomes through personalized hemodynamic analysis and optimized prosthetic valve orientation.

## Conflict of interest

The authors declare no competing interests.

## Figures and Tables

**Fig. 9:**
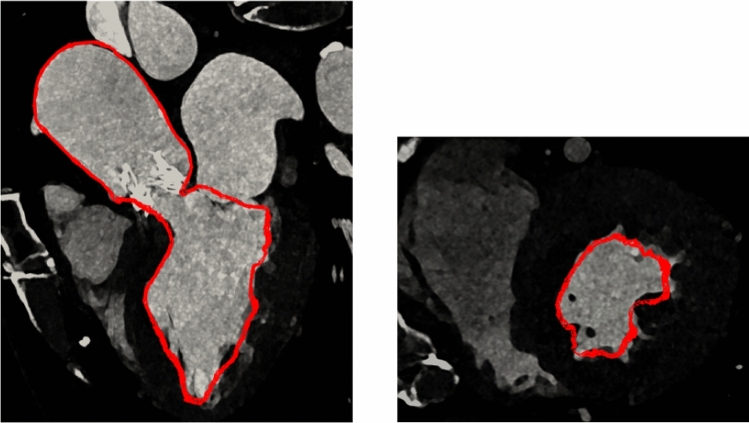
Visual validation of boundary deformation at end-systole. Left: three-chamber (3CH) view. Right: short-axis (SAX) view. The grayscale background shows the original CT image, and the red contours represent the deformed surface geometry obtained from the registration-based motion extraction

**Fig. 10:**
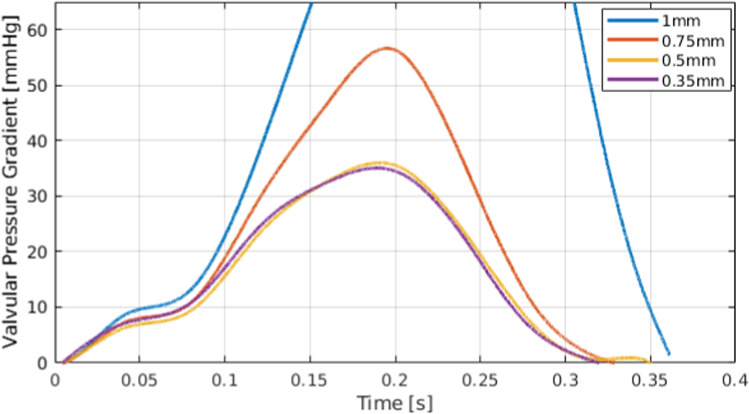
Time history of the valvular pressure drop for the four different grid resolutions. For visibility reasons, the Y-axis range is capped to 65 [mmHg], even though the maximum value of the coarse grid is at 110 [mmHg]

**Fig. 11:**
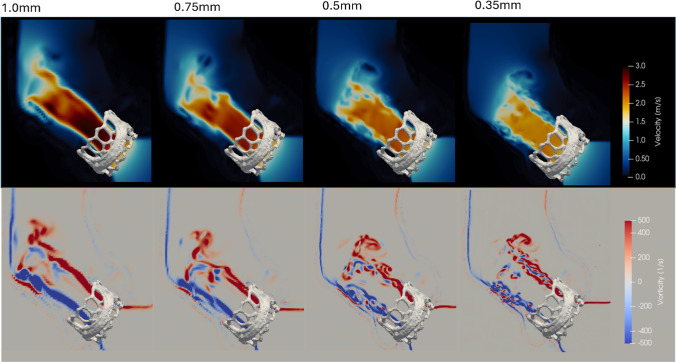
Visualization of velocity magnitude (upper row) and out-of-plane vorticity (lower row) for four different levels of background grid resolution during maximum flow acceleration

**Fig. 12:**
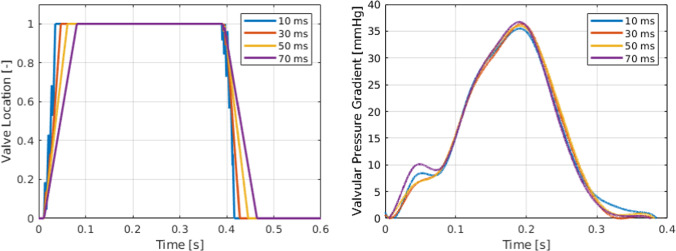
Sensitivity analysis of valve opening and closing time duration. Left: valve position as a function of time for the four evaluated time durations. Right: Resulting pressure gradient over the valve for the time durations

**Fig. 13:**
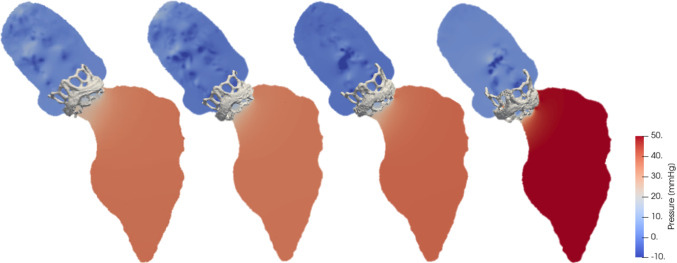
Contour plot of static pressure relative to the ascending aortic outlet pressure. From left to right: baseline case, translated 6 mm ventricularly, rotated 14°, and rotated 27°

## Data Availability

No datasets were generated or analysed during the current study.

## Appendix A

### A1: Overlay of CT images and deformed geometries

To qualitatively assess the accuracy of the boundary deformation relative to the CT-derived anatomy, we generated overlays of the deformed surface geometry and the original CT images at end-systole, see Fig. 9. This is where the structure is at its most deformed state. Two standard cardiac views are shown: a three-chamber (3CH) long-axis view and a short-axis (SAX) view. These comparisons demonstrate that the registration-based motion extraction produces geometries that remain consistent with the underlying CT data throughout the cardiac cycle.

### A2: Solution verification of grid resolution

To ensure that the background grid size did not affect the results, a grid convergence test was performed. We assessed four different grid cases, where each grid had three levels of locally refined grid, and with a refinement factor of four between each level. The fine-level grid resolutions for the four grid cases were 1.0 mm (Coarse), 0.75 mm (Medium), 0.50 mm (Fine) and 0.35 mm (Very Fine). For reference, the in-plane resolution of the cardiac CT data was 0.39 mm and slice thickness 0.3 mm.

The time history of the valvular pressure drop is shown in Fig. 10, and the absolute and relative pressure difference with respect to the 0.35 mm grid are quantified in Table 2.

**Table 2:** Absolute and relative differences in maximum valvular pressure drop with respect to the 0.35 mm grid

Grid case	Peak pressure drop [mmHg]	|∆p_035| [mmHg]	|∆p_035| [%]
Coarse 1 mm	110.2	75.1	213%
Medium 0.75 mm	56.6	21.5	61.2%
Fine 0.5 mm	36.0	0.9	2.6%
Very fine 0.35 mm	35.1	–	–

The coarse 1-mm grid yielded peak valvular pressure drop with over 110 mmHg, while the medium 0.75-mm grid yielded 56 mmHg, compared to the 35.9 mmHg and 35.0 mmHg from the fine and very fine grid. This large difference can be attributed to the effect of the grid-dependent delta-function which will reduce the effective orifice area of the valve. With a coarser grid, the effective orifice area will be reduced, which in turn will increase the valvular pressure drop. In practice, this effect will always be present but will reduce to zero under grid refinement.

To further assess the grid resolution, a slice plane through the valve and ascending aorta was used to visualize the velocity magnitude and vorticity component normal to the plane, see Fig. 11. As can be seen, the velocity magnitude is larger in the coarse and medium grids, as a direct consequence of the smaller effective orifice area in the valve, while the fine and very fine grid present similar results. The fine and very fine grid resolutions also capture the vortex shedding in the valve, as small rotating and counter-rotating vortices can be observed, which is not as discernible in the coarse and medium grids.

As there were only subtle differences between the fine and very fine grid resolutions, it was concluded that the fine grid resolution of 0.5 mm provided a good trade-off between accuracy and compute time.

### A3: Sensitivity analysis of leaflet dynamics

The rate at which the valve leaflets open and close could influence the valvular pressure drop, as a slower opening might reduce the effective valve area available for blood flow, potentially leading to higher pressure gradients. To investigate this, four different opening rates were tested, corresponding to opening times of 10, 30, 50, and 70 ms, consistent with values reported in the literature. Figure 12 shows the leaflet positions and the resulting pressure gradients for each opening duration. The peak pressure gradient was largely unaffected by the valve opening time. During valve opening, minor differences were observed in the slowest case (70 ms). In this case, the delayed opening resulted in a smaller effective orifice area for the current flow rate, causing a slight temporary increase in the pressure drop. This effect then disappeared when the valve was fully open. Based on this analysis, both the valve opening and closing durations were set to 50 ms.

### A4: Static pressure fields at peak flow rate

To complement the velocity field visualizations in the main text, we here present static pressure fields at peak systolic flow (Fig. 13). Pressures are shown relative to the outlet reference pressure, which highlights the elevated left ventricular pressure required to drive flow through the valve prosthesis. The pressure distribution illustrates the transvalvular gradient across the valve and demonstrates that pressure recovers rapidly downstream in the ascending aorta.

Localized regions of elevated pressure are not observed in the ascending aorta, consistent with the fact that the flow jet primarily generates dynamic rather than static pressure effects. Instead, the differences between implantation scenarios are most clearly reflected in the overall transvalvular drop, with the 27° rotation case exhibiting the highest left ventricular pressures. Together with the velocity plots, these fields highlight that the primary hemodynamic burden arises across the valve itself.
